# Combined Impact of Heart Rate Sensor Placements with Respiratory Rate and Minute Ventilation on Oxygen Uptake Prediction

**DOI:** 10.3390/s24165412

**Published:** 2024-08-21

**Authors:** Zhihui Lu, Junchao Yang, Kuan Tao, Xiangxin Li, Haoqi Xu, Junqiang Qiu

**Affiliations:** 1 School of China Football Sports, Beijing Sport University, Beijing 100084, China; 2022210559@bsu.edu.cn; 2 Exercise Science School, Beijing Sport University, Beijing 100084, China; 1004320180291@bsu.edu.cn (J.Y.); 2023210294@bsu.edu.cn (X.L.); xuhaoqi200122@outlook.com (H.X.); 3 School of Sports Engineering, Beijing Sport University, Beijing 100084, China; taokuan@bsu.edu.cn; 4 Key Laboratory of Exercise and Physical Fitness, Ministry of Education, Beijing Sport University, Beijing 100084, China; 5 Beijing Sports Nutrition Engineering Research Center, Beijing 100084, China

**Keywords:** oxygen uptake prediction, heart rate, machine learning, electrocardiography, photoplethysmography

## Abstract

Oxygen uptake ($\dot{V}O_{2}$) is an essential metric for evaluating cardiopulmonary health and athletic performance, which can barely be directly measured. Heart rate (HR) is a prominent physiological indicator correlated with $\dot{V}O_{2}$ and is often used for indirect $\dot{V}O_{2}$ prediction. This study investigates the impact of HR placement on $\dot{V}O_{2}$ prediction accuracy by analyzing HR data combined with the respiratory rate (RESP) and minute ventilation ($\dot{V}E$) from three anatomical locations: the chest; arm; and wrist. Twenty-eight healthy adults participated in incremental and constant workload cycling tests at various intensities. Data on $\dot{V}O_{2}$, RESP, $\dot{V}E$, and HR were collected and used to develop a neural network model for $\dot{V}O_{2}$ prediction. The influence of HR position on prediction accuracy was assessed via Bland–Altman plots, and model performance was evaluated by mean absolute error (MAE), coefficient of determination (R^2^), and mean absolute percentage error (MAPE). Our findings indicate that HR combined with RESP and $\dot{V}E$ ($\dot{V}O_{2HR+RESP+\dot{V}E}$) produces the most accurate $\dot{V}O_{2}$ predictions (MAE: 165 mL/min, R^2^: 0.87, MAPE: 15.91%). Notably, as exercise intensity increases, the accuracy of $\dot{V}O_{2}$ prediction decreases, particularly within high-intensity exercise. The substitution of HR with different anatomical sites significantly impacts $\dot{V}O_{2}$ prediction accuracy, with wrist placement showing a more profound effect compared to arm placement. In conclusion, this study underscores the importance of considering HR placement in $\dot{V}O_{2}$ prediction models, with RESP and $\dot{V}E$ serving as effective compensatory factors. These findings contribute to refining indirect $\dot{V}O_{2}$ estimation methods, enhancing their predictive capabilities across different exercise intensities and anatomical placements.

## 1. Introduction

Oxygen uptake ($\dot{V}O_{2}$), the quantity of oxygen consumed by the body, serves as a vital indicator of both energy expenditure [1] and exercise intensity [2]. It also assesses the body’s capacity to ingest and utilize oxygen, which is directly associated with key health metrics such as fitness performance [3,4,5,6] and cardiorespiratory health [7,8,9]. Additionally, $\dot{V}O_{2}$ plays a crucial role in the prevention and management of various diseases, including heart failure and chronic obstructive pulmonary disease [10,11,12]. Traditionally, $\dot{V}O_{2}$ has been measured directly using a gas metabolism analyzer system, which is impractical for non-laboratory settings due to its high cost and operational complexity. Consequently, the indirect measurement of $\dot{V}O_{2}$ is critical in daily monitoring, offering valuable insights into exercise assessment and clinical symptom identification.

In the indirect prediction of $\dot{V}O_{2}$, heart rate (HR), which is easily accessible, is used as the key variable due to its strong correlation with $\dot{V}O_{2}$ [13,14,15,16]. HR is primarily measured through electrocardiography (ECG) and photoplethysmography (PPG). While ECG is regarded as the gold standard [17,18], its requirement for electrode placement renders it less convenient for monitoring physical activities [19,20]. On the other hand, PPG provides greater portability and ease of use by detecting blood volume that changes optically at the skin surface [21]. Consequently, PPG signal acquisition for HR monitoring is more widely utilized in everyday health surveillance, particularly with wearable devices, due to its operational simplicity and ease of wearability. Therefore, it is essential to validate whether HR from PPG is as effective as that from the ECG, given their importance in the indirect prediction of $\dot{V}O_{2}$.

In light of growing interest in the precision of indirect $\dot{V}O_{2}$ measurement methods, the FLEX-HR method has demonstrated efficacy in predicting $\dot{V}O_{2}$ within free-living settings, thereby enhancing the HR-$\dot{V}O_{2}$ relationship. This method, which aims to enhance the HR-$\dot{V}O_{2}$ relationship [22,23], requires participants to undertake direct $\dot{V}O_{2}$ measurement across various activities. These assessments are essential for making necessary adjustments to the HR-$\dot{V}O_{2}$ curve based on intensity variations, although they may restrict its practical application. Consequently, the inclusion of additional input features has been explored, revealing that integrating other signals with HR, such as acceleration and exercise duration—common variables in exercise—enhances the accuracy of $\dot{V}O_{2}$ estimations in low-intensity activities [24,25]. However, these factors do not provide insights into physical effort and are less effective for repetitive cyclic movements. Furthermore, respiratory parameters strongly correlated with $\dot{V}O_{2}$, such as respiratory rate (RESP) and minute ventilation ($\dot{V}E$), have attracted attention. These parameters reflect metabolically induced changes in the body during exercise. Although previous research on these respiratory signals showed promise in predicting $\dot{V}O_{2}$ [26], collecting these signals together with HR from the same source can lead to homologous data errors, potentially undermining the reliability of the results.

In parallel with the exploration of additional physiological signals, this research investigates alternative predictive models to elucidate further information embedded within HR or its combination with other physiological parameters. Historically, linear regression has been commonly employed to predict oxygen uptake [1,27,28]. However, this method has significant limitations in predicting $\dot{V}O_{2}$ across varying intensities. Currently, machine learning has been recognized as a means to enhance the accuracy of $\dot{V}O_{2}$ predictions [29]. For instance, models such as random forest and long short-term memory (LSTM) have utilized readily available inputs like HR, RESP, and $\dot{V}E$ to predict $\dot{V}O_{2}$ fluctuations during low-intensity exercises or daily activities [30,31,32,33,34], highlighting the potential application of these algorithms in wearable devices and fitness equipment. However, it is noteworthy that most of the exercise data involved predominantly pertain to low- and moderate-intensity steady-state exercises, neglecting variations in intensity and type of exercise [23], which could lead to underestimations or overestimations of $\dot{V}O_{2}$. Moreover, these studies utilize ECG for HR acquisition, overlooking potential discrepancies that could arise from different HR signal sources, potentially resulting in deviations in the outcomes.

With the increasing use of PPG in wearable devices for daily health monitoring, establishing its accuracy in reflecting $\dot{V}O_{2}$ is critical for enhancing the reliability of both fitness and clinical assessments. Therefore, this research seeks to determine if HR measurements from PPG are as reliable as those from ECG for the indirect estimation of $\dot{V}O_{2}$. Additionally, this study integrates different HR sources with physiological signals such as RESP and $\dot{V}E$ into machine learning algorithms to refine $\dot{V}O_{2}$ predictions, accounting for varying exercise intensities and types. The findings will contribute to the optimization of non-invasive $\dot{V}O_{2}$ monitoring technologies, with significant implications for health evaluation and fitness performance analysis.

## 2. Materials and Methods

### 2.1. Participants

Twenty-eight healthy, well-trained participants (16 males and 12 females) with no known cardiovascular, musculoskeletal, respiratory, or metabolic diseases were enrolled in this study. Participants with any of these diseases or related symptoms were excluded. All participants were thoroughly informed about the experimental procedures and potential risks. Each exercise test was conducted at least two hours postprandial, with no alcohol or caffeine intake and no vigorous exercise in the preceding 24 h. Female participants were advised to avoid completing the test during their menstrual period. All exercise tests were spaced at least 48 h apart. Participants wore lightweight sportswear during the tests. All procedures were approved by the Research Ethics Committee of Beijing Sport University (reference number 2024016H).

### 2.2. Procedure

In this study, each participant completed six cycling exercise tests, including one incremental exercise test and five constant workload tests of varying intensities. Anthropometric measurements, including height, weight, and body composition, were recorded prior to the initial exercise test. For the first test, the cycle ergometer’s handlebar angle and seat height were adjusted to ensure an upright riding position. Specifically, the seat height was adjusted so that the participants’ knees were slightly bent at the lowest pedal position. These settings were recorded and consistently maintained for all subsequent tests.

During each exercise test, participants wore a gas metabolism analyzer and three HR monitoring devices. After donning the devices, participants remained seated for five minutes to record resting breath gas and HR data. This was followed by a structured warm-up session, during which participants cycled at 80 W with a self-selected pedal speed for five minutes. During the exercise tests, participants maintained a pedal speed of 60 ± 5 rpm, receiving feedback from the ergometer’s rpm display and a metronome. Upon completing the exercise, participants rested for ten minutes to facilitate the collection of excess post-exercise oxygen consumption (EPOC) data.

The incremental test began with a load of 50 W for four minutes, followed by an increase of 30 W every minute until exhaustion. Exhaustion was confirmed when any three of the following criteria were met: (1) a $\dot{V}O_{2}$ increment of ≤2.1 mL/(kg·min); (2) post-exercise blood lactate ≥ 8.0 mmol/L; (3) respiratory exchange ratio ≥ 1.1; and (4) maximum HR ≥ 100% HRmax (208 − 0.7 × age) [2]. The loads for the subsequent low, medium, and high-intensity tests were determined based on the peak power output (PPO) in the incremental test. The order of the constant load tests was randomized, and participants were required to continue for ten minutes or until exhaustion. The laboratory environment was controlled, with a temperature of approximately 20–25 °C and relative humidity of 50–60%.

### 2.3. Data Collection

The body composition of the subjects was determined using a digital dual-energy X-ray absorptiometry (DXA) device (iDxa, General Electric Company, Fairfield, CT, USA). Respiratory gases during the incremental and constant workload exercise tests were analyzed using a spiroergometry system (MetaMax 3B, Cortex Biophysic, Leipzig, Germany) for breath-by-breath measurements, including $\dot{V}O_{2}$, RESP, and $\dot{V}E$. Calibration of air pressure, gas (standard gas concentrations of O_2_ 15.00%, CO_2_ 5.00%), and volume flow (using a 3 L syringe) was conducted according to the manufacturer’s instructions before each exercise test. Fingertip blood samples were collected immediately after the incremental exercise test for blood lactate analysis. All exercise tests were performed on a cycle ergometer (839E, Monark Exercise AB, Vansbro, Sweden).

The HR measurement devices used in this study were all from Polar (Polar Electro Oy, Kempele, Finland), specifically, the H10 chest strap (using ECG), the Vantage V wrist device, and the Verity Sense armband (both using PPG). Each device provides HR data every second using proprietary algorithms to filter and process the detected heartbeats.

1. The Polar H10 uses a 1000 Hz sample rate to gather data for internal sensor calculations and algorithms. Subsequently, the sensor outputs the processed data at a 130 Hz sampling rate. The H10 transmits HR data once per second;
2. The Polar Vantage V utilizes PPG and provides HR samples at a rate of 1 Hz. According to Polar, the internal sampling rate of the Vantage V is considerably higher, and the 1 Hz HR data is derived from this higher-rate sampling;
3. The Polar Verity Sense utilizes PPG, with a sampling rate of 135 Hz and a resolution of 22 bits.

**Figure 1:**
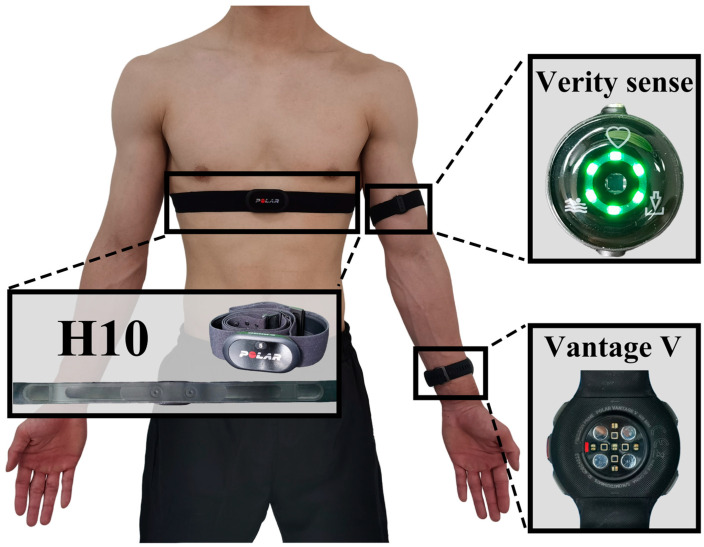
Position of sensor wearing.

The Polar H10 was designated as the standard HR monitor in this study. The chest strap electrodes were moistened before being secured to the participants’ chests during the xiphoid process. The Vantage V was worn on the subject’s non-dominant wrist, at least a finger’s width from the wrist bone. The Verity Sense was worn on the bicipital muscle in the upper arm, with the armband on the subjects’ non-dominant sides, ensuring the sensor was placed firmly against the skin. These three devices were replaced on the participants’ body parts, as shown in Figure 1. During this experiment, care was taken to ensure that the devices were worn securely and comfortably. Following the manufacturer’s recommendations, all data were transferred and synchronized via Bluetooth. The raw data from the exercise sessions, including timestamps and HR data from the three devices, were then exported through Polar Flow.

### 2.4. Data Processing and Model Construction

#### 2.4.1. Data Standardization

In this study, the feature data used as inputs to the neural network were taken from the Polar H10 chest strap (i.e., HR, RESP and $\dot{V}E$). Considering the dimensional differences between various features, especially the impact of these differences on the training convergence speed during the gradient descent method in neural network training, this study uses the Z-score normalization method to standardize the input and output feature data to have a mean of 0 and a variance of 1. The formula for the Z-score normalization method is as follows:

(1) $$Z=\frac{X-\mu }{\sigma }$$

where X denotes the original data values; μ and σ represent the mean and standard deviation of the data, respectively.

#### 2.4.2. Building of Backpropagation Neural Network (BPNN)

**Figure 2:**
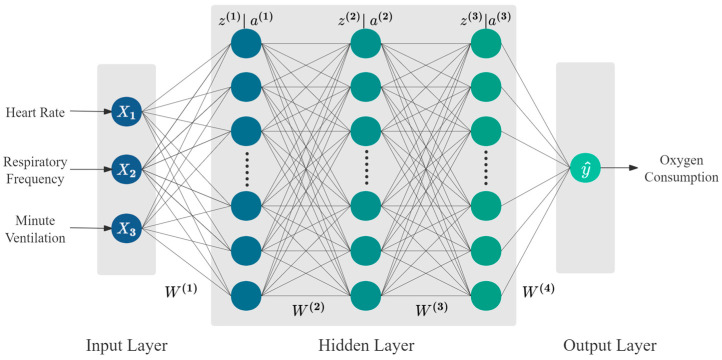
The structure of backpropagation neural network.

Backpropagation Neural Network (BPNN) is one of the fundamental methods in deep learning, with a wide range of applications. From natural language processing and computer vision to speech recognition and bioinformatics, BPNN has become an essential tool for addressing various complex problems. A BPNN is a type of feedforward neural network that trains weights and biases using the backpropagation algorithm to minimize prediction error. In this study, a BPNN with one input layer, three hidden layers, and one output layer is constructed to capture the nonlinear mapping between physiological signals and $\dot{V}O_{2}$, as shown in Figure 2.

To investigate whether there is a strong mapping relationship between different physiological signals and $\dot{V}O_{2}$, four types of inputs were considered when constructing the BPNN in this study as follows [35]:

1. From heart rate (HR) only, named $\dot{V}O_{2HR}$;
2. From heart rate (HR) and respiratory rate (RESP), named $\dot{V}O_{2HR+RESP}$;
3. From heart rate (HR) and minute ventilation ($\dot{V}E$) named $\dot{V}O_{2HR+\dot{V}E}$;
4. From heart rate (HR), respiratory rate (RESP), and minute ventilation ($\dot{V}E$), named $\dot{V}O_{2HR+RESP+\dot{V}E}$.

It should be noted that for the four different types of input features, this model was built using the same architecture, only changing the dimension of the input layer features. As shown in Figure 2, the BPNN achieves model training through two processes: forward propagation and backpropagation, with the principles and methodology described as follows:

(1) Forward Propagation

First, the normalized physiological signal data are fed into the input layer of the BPNN as the input feature vector $x={\left[x_{1},x_{2},\ldots ,x_{i}\right]}^{T}$ (i = 1, 2, or 3). Then, the input feature vector x is computed and input into the first hidden layer, yielding the linear combination vector $z^{\left(1\right)}$ and the activation vector $a^{\left(1\right)}$ of the first hidden layer as follows:

(2) $$z^{\left(1\right)}=W^{\left(1\right)}x+b^{\left(1\right)}$$

(3) $$a^{\left(1\right)}=f(z^{\left(1\right)})$$

where $W^{\left(1\right)}$ and $b^{\left(1\right)}$ are the weight matrix and bias vector for the first hidden layer, respectively, and f(⋅) is the activation function ReLU, defined as f(x)=max(0,x).

Between hidden layers, similarly, for the l-th hidden layer (l = 2, 3), based on the activation vector $a^{\left(1\right)}$ from the first hidden layer, the linear combination vector $z^{\left(l\right)}$ and the activation vector $a^{\left(l\right)}$ are computed as follows:

(4) $$z^{\left(l\right)}=W^{\left(l\right)}a^{\left(l-1\right)}+b^{\left(l\right)}$$

(5) $$a^{\left(l\right)}=f(z^{\left(l\right)})$$

where $W^{\left(l\right)}$ and $b^{\left(l\right)}$ are the weight matrix and bias vector for the l-th layer, respectively.

Finally, the activation vector $a^{\left(3\right)}$ from the last hidden layer is input into the output layer to obtain the linear combination vector $z^{\left(4\right)}$ and the final predicted value $\hat{y}$, computed as follows:

(6) $$z^{\left(4\right)}=W^{\left(4\right)}a^{\left(3\right)}+b^{\left(4\right)}$$

(7) $$\hat{y}=z^{\left(4\right)}$$

where $W^{\left(4\right)}$ and $b^{\left(4\right)}$ are the weight matrix and bias vector for the output layer, respectively.

After obtaining the prediction result from the output layer, this study uses the Mean Squared Error (MSE) to measure the difference between the predicted values and the true values. The definition of MSE is as follows:

(8) $$MSE=\frac{1}{n}\sum_{i=1}^{n}{\left({\hat{y}}_{i}-y_{i}\right)}^{2}$$

where n is the total number of samples; ${\hat{y}}_{i}$ is the predicted value of the i-th sample, and $y_{i}$ is the true value of the i-th sample;

(2) Backward Propagation

After forward propagation and loss calculation, the predicted values of the model are obtained, as well as the error between the predicted values and the actual values. Then, the backpropagation algorithm needs to calculate the gradient of the loss function with respect to each parameter (weights and bias) in the network, which represents the rate of change in the loss function with respect to each parameter in the parameter space. By calculating the obtained gradient information, the backpropagation algorithm adjusts the weights and biases in order to reduce the value of the loss function, which results in a more accurate model prediction accuracy.

The error term $\delta^{\left(4\right)}$ for the output layer is calculated as follows:

(9) $$\delta^{\left(4\right)}=(\hat{y}-y)$$

And the corresponding gradients of the weights and biases are as follows:

(10) $$\frac{\partial L}{\partial W^{\left(4\right)}}=\delta^{\left(4\right)}a^{{\left(3\right)}^{T}},\frac{\partial L}{\partial b^{\left(4\right)}}=\delta^{\left(4\right)}$$

For the l-th hidden layer (l = 3, 2, 1), the error term $\delta^{\left(l\right)}$ is calculated as follows:

(11) $$\delta^{\left(l\right)}={\left(W^{\left(l+1\right)}\right)}^{T}\delta^{\left(l+1\right)}⊙f^{\prime }(z^{\left(l\right)})$$

where ⊙ denotes the Hadamard product, and $f^{\prime }$ is the derivative of the ReLU activation function.

The corresponding gradients of the weights and biases are as follows:

(12) $$\frac{\partial L}{\partial W^{\left(l\right)}}=\delta^{\left(l\right)}a^{{\left(l-1\right)}^{T}},\frac{\partial L}{\partial b^{\left(l\right)}}=\delta^{\left(l\right)}$$

Finally, the weights and biases of both the output layer and the hidden layers are updated using gradient descent as follows, and η is the learning rate:

(13) $$W^{\left(l\right)}=W^{\left(l\right)}-\eta \frac{\partial L}{\partial W^{\left(l\right)}},b^{\left(l\right)}=b^{\left(l\right)}-\eta \frac{\partial L}{\partial b^{\left(l\right)}}$$

#### 2.4.3. Training Parameter Setting

In this study, all models were implemented in Python (version 3.6, Python Software Foundation, Beaverton, OR, USA). Allocate 80% of the data to the training set, 10% to the test set, and 10% to the validation set. This model was compiled using the Adam optimizer and the MSE loss function for 100 training epochs, with the batch size set to 32. Optimal parameters and node combinations were identified through trial and error.

### 2.5. Statistical Analysis

The results are represented as means ± standard deviation (SD). The concordance correlation coefficient (CCC) was calculated to evaluate the agreement between the devices, with 95% confidence intervals (CI) provided to assess the precision of the estimates. Deviations between measured and predicted values were evaluated using linear regression. The correlation between measured and predicted values was determined using the Pearson correlation coefficient. Additionally, a Bland–Altman plot was used to assess the agreement between measured and predicted data. The predictive accuracy of the models for the entire dataset and each exercise intensity was assessed using mean absolute error (MAE), mean absolute percentage error (MAPE), and percentage error (% error). The maximum acceptable error limit was set at 200 mL/min, which represents the typical noise during exercise [31]. All statistical analyses were performed with GraphPad Prism 10 (GraphPad Software, La Jolla, CA, version 10.1.2) and Python (version 3.6, Python Software Foundation).

## 3. Results

### 3.1. Participants’ Characteristics and Exercise Responses

The characteristics of the participants are detailed in Table 1. Each participant underwent an incremental load test to establish baseline exercise intensity levels, followed by five experimental trials (a total of 140 trials). This study aimed to investigate the effect of HR sensor positions on $\dot{V}O_{2}$ prediction and to determine whether differences existed across various exercise intensities. Based on participants’ responses during the incremental test, five exercise intensity levels were selected for each participant. These levels correspond to the participants’ individual $\dot{V}O_{2}max$ as follows: low-intensity steady-state exercise at 45% $\dot{V}O_{2}max$; moderate-intensity steady-state exercise at 60% $\dot{V}O_{2}max$; heavy-intensity steady-state exercise at 75% $\dot{V}O_{2}max$; very-heavy-intensity exhaustive exercise at 95% $\dot{V}O_{2}max$; and severe-intensity exhaustive exercise at 110% $\dot{V}O_{2}max$. In the tests at 45% to 75% $\dot{V}O_{2}max$, participants completed 10 min of cycling exercise. In the tests at 95% and 110% $\dot{V}O_{2}max$, due to the higher exercise intensity, steady-state exercise could not be achieved; therefore, the duration was set to exhaustion. After excluding anomalous data due to poor sensor contact or connection issues, a total of 160,429 valid data points were retained. Additionally, the duration of recording for each test in the final dataset was standardized across all trials.

### 3.2. Descriptive of Heart Rate Measurements: H10, Vantage and Verity Sense

**Figure 3:**
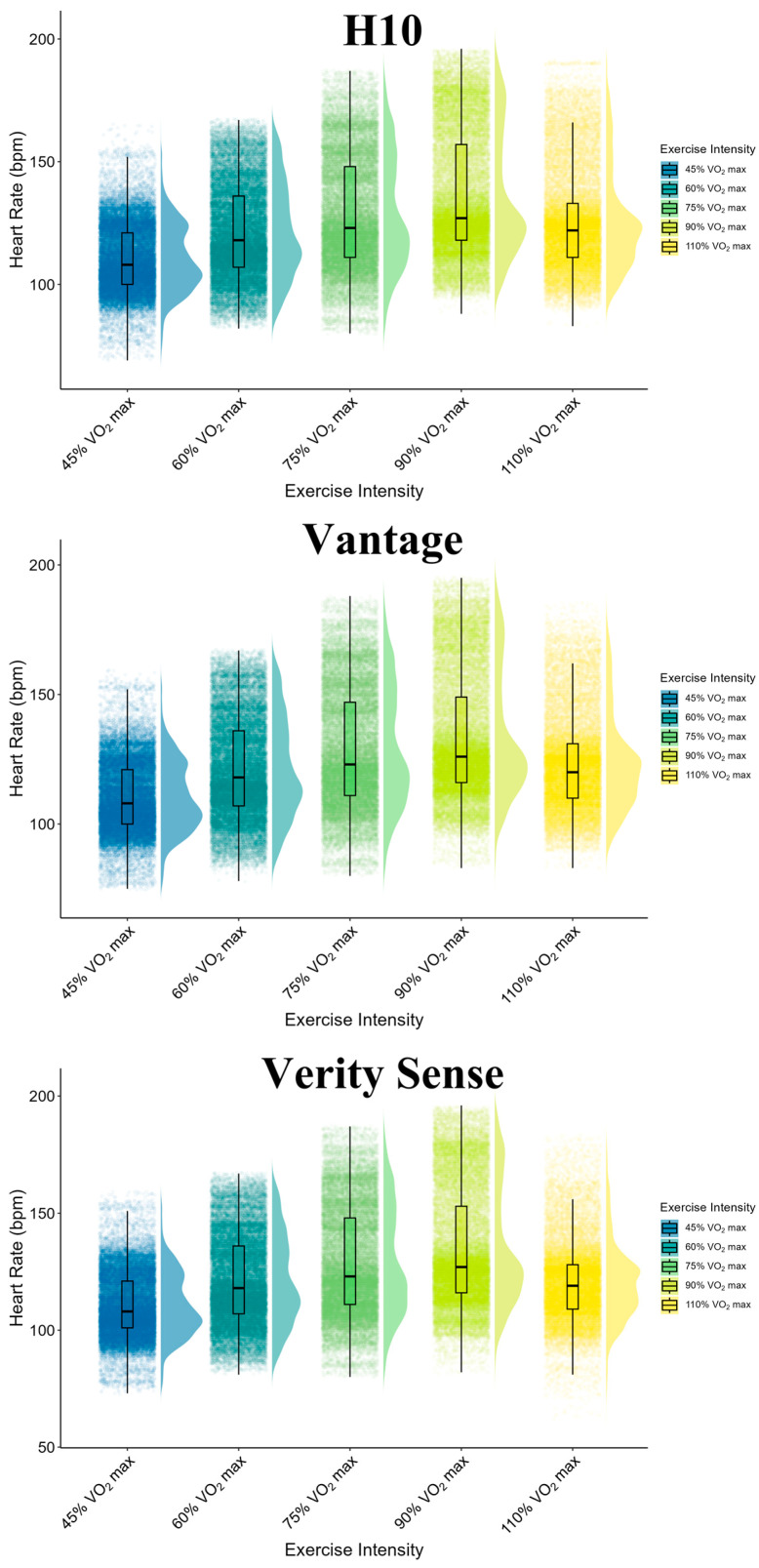
A plot of HR measurements for H10, Vantage, and Verity Sense. Different colors represent each exercise intensity level. The black line in the middle of the box represents the median HR; the edges of the box represent the 25th and 75th percentiles of HR, and the whiskers show the range of HR. A split-half violin plot with density plots overlaid in the background illustrates the distribution density of the HR.

HR data were collected from the H10, Vantage, and Verity Sense devices, resulting in a total of 160,429 paired data points sampled at a frequency of 1 Hz. Missing data points were excluded from the analysis. The data were collected across five different exercise intensities. As exercise intensity increased, both the mean and standard HR error rose. However, at 90% and 110% $\dot{V}O_{2}max$, the proportion of HR readings during the EPOC phase was higher due to the shorter exercise duration. The overall characteristics of the collected data are illustrated in Figure 3.

### 3.3. Accuracy of Different Sensor Positions: Vantage vs. Verity Sense

HR data were collected from the H10, Vantage, and Verity Sense devices, resulting in a total of 160,429 paired data points sampled at a frequency of 1 Hz. Missing data points were excluded from the analysis. The data were collected across five different exercise intensities. As exercise intensity increased, both the mean and standard error of HR rose. However, at 90% and 110% $\dot{V}O_{2}max$, the proportion of HR readings during the EPOC phase was higher due to the shorter exercise duration. The overall characteristics of the collected data are illustrated in Figure 1. The HR accuracy of the Vantage and Verity Sense devices across varied exercise intensities is shown in Table 2. At lower intensities (45–75% $\dot{V}O_{2}max$), both devices demonstrated minimal MAE with the Vantage and Verity Sense recording values of 2.59 ± 2.40 and 1.95 ± 2.00 at 45% $\dot{V}O_{2}max$, 2.19 ± 2.18 and 1.83 ± 2.75 at 60% $\dot{V}O_{2}max$ and 1.92 ± 2.09 and 1.52 ± 1.85 at 75% $\dot{V}O_{2}max$, respectively. These results were corroborated by high CCC and Pearson correlation coefficients, exceeding 0.95 for both devices.

At higher intensities (95% and 110% $\dot{V}O_{2}max$), the MAE and variability increased significantly. At 95% $\dot{V}O_{2}max$, the Vantage exhibited an MAE of 3.62 ± 7.26, while the Verity Sense showed 2.83 ± 6.06. The respective CCC values were 0.85 (95% CI, 0.85–0.85) and 0.91 (95% CI, 0.91–0.91). Notably, at 110% $\dot{V}O_{2}max$, the performance of the Verity Sense declined sharply with an MAE of 4.79 ± 10.17 and a CCC of 0.54 (95% CI, 0.53–0.54), compared to Vantage’s MAE of 3.49 ± 6.56 and CCC of 0.81 (95% CI, 0.81–0.82). This suggests that the Verity Sense performs better at lower-intensity exercises, while both devices exhibit greater errors at higher-intensity levels.

### 3.4. Impact of Sensor Placements on $\dot{V}O_{2}$ Prediction across Varied Exercise Intensities

To verify the impact of different HR sensor positions on $\dot{V}O_{2}$ prediction accuracy across various exercise intensities, neural network models were constructed using data from these different intensities. Table 3 presents the results of the modeling based on different exercise intensity data and illustrates the impact of HR sensor placements on the accuracy of the $\dot{V}O_{2}$ prediction model.

The prediction results of the exercise at 45% and 60% $\dot{V}O_{2}$max showed weaker correlation and higher MAE in the $\dot{V}O_{2HR}$ and $\dot{V}O_{2HR+RESP}$. Meanwhile, in the model incorporating $\dot{V}E$ as an input, the HR from different positions did not significantly affect the predictive ability. The substitution of HR from Vantage and Verity Sense maintained the error within 200 mL/min, and the MAPE was below 15% in both the $\dot{V}O_{2HR+\dot{V}E}$ and $\dot{V}O_{2HR+RESP+\dot{V}E}$ models. The $\dot{V}O_{2HR+RESP+\dot{V}E}$ model configuration showed the lowest MAE and MAPE (68.46 mL/min, 7%) when HR was replaced with Verity Sense at 45% $\dot{V}O_{2}$max, indicating strong predictive ability.

In contrast, the models based on data with higher-intensity exercises (95% $\dot{V}O_{2}$max and 110% $\dot{V}O_{2}$max) demonstrated different predictive results, showing higher MAE and MAPE. Moreover, the HR data from the Vantage and Verity Sense significantly diminished the correlation at these high intensities, especially for the $\dot{V}O_{2HR}$ model, which only included one variable. This result suggests that the HR data from the Vantage and Verity Sense under high-intensity exercises cannot be used as valid predictive inputs for predicting $\dot{V}O_{2}$.

Among these five tests, the model trained on 75% $\dot{V}O_{2}$max exercise all demonstrated the best fit and prediction accuracy, with the highest correlations for models with different combinations of physiological parameters. Additionally, the HR inputs of Vantage and Verity Sense similarly maintained a low MAE and MAPE.

### 3.5. Comprehensive Oxygen Consumption Prediction Accuracy with Heart Rate Positioning: Overall Exercise Intensities

**Figure 4:**
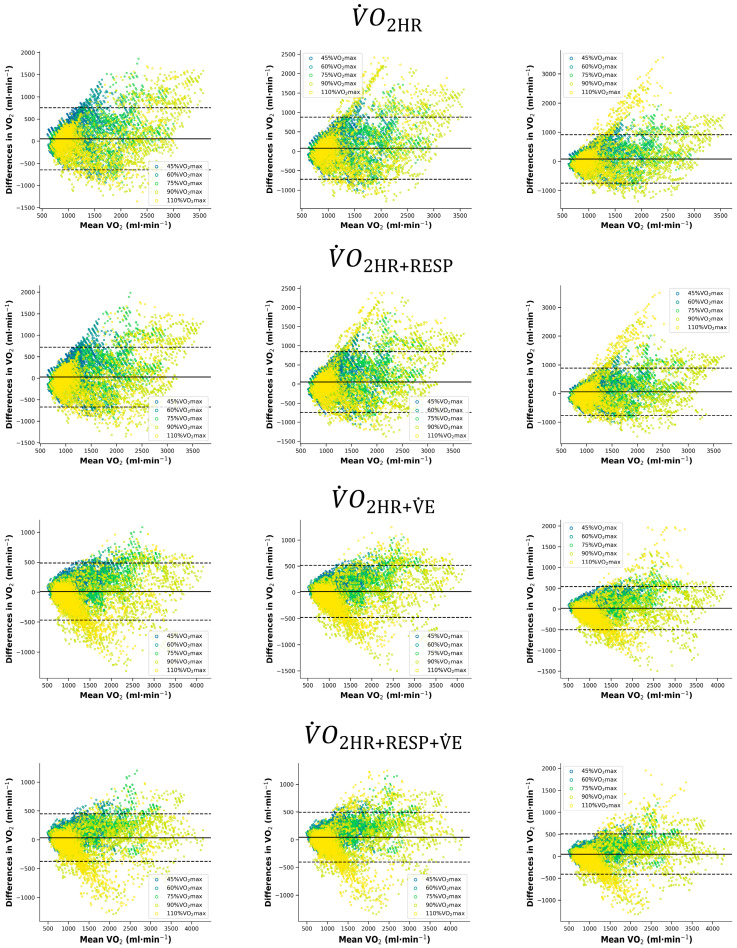
Bland–Altman analysis for the measured $\dot{V}O_{2}$ and predicted $\dot{V}O_{2}$ in different models. The solid line represents the prediction bias, and the dashed line represents 95% limits of agreement. In A and B, a different color represents different exercise intensities. The HR data of the left panel were from H10; the middle panel was from Vantage, and the right panel was from Verity Sense.

When using a dataset encompassing all exercise intensities, modelling was performed with four combinations of inputs: HR; HR + RESP; HR + $\dot{V}E$; and HR + RESP + $\dot{V}E$. Table 4 presents the analysis of four different models and HR devices on the accuracy of predicting $\dot{V}O_{2}$, including R^2^, MAE, MAPE, and the percentage increase in error. Figure 4 illustrates the agreement between measured $\dot{V}O_{2}$ and predicted $\dot{V}O_{2}$ using a Bland–Altman plot.

As shown in Table 4, adding information to the $\dot{V}O_{2HR}$ model significantly increased the accuracy of the estimation. It can be observed that the parameter combinations of $\dot{V}O_{2HR+RESP+VE}$ and $\dot{V}O_{2HR+VE}$ have the highest prediction accuracy, both within 200 mL/min, in which $\dot{V}O_{2HR+RESP+VE}$ generated the best predictions with the highest R^2^ and the lowest MAE and MAPE among the four models, suggesting that HR combined with RESP and $\dot{V}E$ can enhance prediction accuracy compared to the other combinations.

When the input data covered all exercise intensities, the predictive accuracy of the models decreased to some extent after replacing the HR with the data collected through Vantage and Verity Sense. However, this decreasing trend was more pronounced in $\dot{V}O_{2HR}$ and $\dot{V}O_{2HR+RESP}$, as represented by the magnitude of the increase in MAE and MAPE. However, when $\dot{V}E$ was included as an input, the predictive accuracy of the different HR sensors increased, with errors remaining within 200 mL/min. Overall, the MAE exceeded 7% in the $\dot{V}O_{2HR}$ and $\dot{V}O_{2HR+RESP}$ models, but the error was reduced in the $\dot{V}O_{2HR+RESP+VE}$ model. The results indicate that, compared to the H10, although substituting HR data from the Vantage and Verity Sense leads to errors, these two HR placements still provide accurate predictions within the multi-physiological parameter model framework.

## 4. Discussion

In this study, we found that the HR from different sensor positions affected the accuracy of $\dot{V}O_{2}$ prediction using BPNN, while this estimation gap could be narrowed by incorporating RESP and $\dot{V}E$ as inputs. This is the first study to investigate the impact of different HR sources on $\dot{V}O_{2}$ prediction. Our findings showed that HR acquisition accuracy was poorer for Vantage and Verity Sense at high-intensity exercises compared to the lower-intensity tests, which led to errors in $\dot{V}O_{2}$ prediction during corresponding intensity exercises. However, in the BPNN, which combined physiological features such as RESP and $\dot{V}E$, HR signals acquired from other than H10 could still accurately estimate the $\dot{V}O_{2}$ values.

With the development of wearable technology, HR and $\dot{V}O_{2}$ are becoming accessible to the public with individual health monitoring. However, this accessibility also poses challenges to the reliability and validity of these monitoring devices and prediction algorithms. The application of these findings is valuable, as different HR devices are increasingly used in $\dot{V}O_{2}$ prediction. Often, the differences in HR extraction are overlooked, with HR sources directly substituted as inputs without specific tuning.

Specifically, $\dot{V}O_{2}$ prediction is typically based on HR, with current research often utilizing HR from ECG signals. While the accuracy of different HR devices has received attention, there is a lack of studies investigating whether HR measurements from other positions can replace ECG in $\dot{V}O_{2}$ prediction. Common HR monitoring is based on PPG signals from the arm and wrist. Thus, this study aimed to explore the impact of different HR measurements on $\dot{V}O_{2}$ prediction by using three HR devices from Polar, a leading brand in the field of exercise HR monitoring; the chest-worn H10, the wrist-worn Vantage, and the arm-worn Verity Sense were chosen.

In the $\dot{V}O_{2}$ prediction model set up with specific exercise intensities, we observed that the influence of Vantage and Verity Sense on $\dot{V}O_{2HR}$ was consistent with their differences in HR measurement accuracy. As exercise intensity increased, the accuracy of HR measurements from both Vantage and Verity Sense decreased, leading to the deviations in $\dot{V}O_{2}$ prediction. The Verity Sense showed slightly better prediction accuracy than the Vantage. However, during severe-intensity exercise, the Verity Sense showed larger measurement errors compared to Vantage (r = 0.82 and 0.57, respectively), which was also reflected in $\dot{V}O_{2}$ values (MAE = 315.36 and 221.55 mL/min, respectively). The potential reason is related to cycling, a lower-limb dominant exercise, which avoids wrist-worn PPG noise associated with arm movements but introduces new interference [36,37,38]. When exercise intensity suddenly increases, the oxygen demand of the leg muscles is higher, leading to prioritized blood flow to the major working muscles and delayed blood flow to the muscles of the wrist and upper arm (HR lag) [39,40], affecting PPG signal acquisition [41]. Furthermore, the need to grip the handlebar to maintain central stability also hinders blood pulse detection, potentially enhancing the difficulty of HR monitoring in the arm [42,43]. Therefore, different HR measurement positions affected $\dot{V}O_{2}$ prediction accuracy. In particular, HR measurement differences at different positions potentially caused larger $\dot{V}O_{2}$ prediction errors during high-intensity exercise.

At 75% $\dot{V}O_{2}$max, the goodness-of-fit for $\dot{V}O_{2HR}$ was the best (R^2^ = 0.74), while it was the poorest at 45% and 110% intensities (R^2^ = 0.27 and 0.35, respectively), which could be explained by the nonlinear relationship between $\dot{V}O_{2}$ and at low- and severe-intensity exercises [1,44]. Thus, to accurately estimate $\dot{V}O_{2}$, it is necessary to incorporate other physiological features [45,46]. Our study found that $\dot{V}E$ and RESP could compensate for the limitations of single HR input in dynamic $\dot{V}O_{2}$ prediction. With the inclusion of $\dot{V}E$ and RESP, the goodness-of-fit at 45% and 110% intensities significantly improved (R^2^ = 0.91 and 0.83, respectively). Furthermore, in models with the Verity Sense as HR substitution, which presented large error, adding $\dot{V}E$ and RESP enhanced prediction ability (R^2^ = 0.75, MAE = 182.67, MAPE = 14.25%), demonstrating the advantage of using multiple physiological signals to enhance $\dot{V}O_{2}$ prediction precision. However, it is interesting to note that the inclusion of RESP in the $\dot{V}O_{2HR+RESP}$ did not improve $\dot{V}O_{2}$ prediction accuracy as much as $\dot{V}E$ [26]. Although RESP is highly sensitive to non-metabolic stressors and may have implications for training and recovery monitoring, its high sensitivity also introduces large individual differences, which fail to significantly improve $\dot{V}O_{2}$ prediction accuracy [47,48,49].

In practical prediction, it is impossible to know exercise intensity without completing the exercise, and the intensity is typically not constant throughout the process. To investigate this contradiction, we also implemented a model with a dataset that includes all exercise intensities. Similarly, the addition of $\dot{V}E$ and RESP improved prediction accuracy and compensated for errors caused by different HR acquisition positions from Vantage and Verity Sense. However, the overall model underperformed the intensity-specific models, possibly due to the differences in exercise durations contributing to various proportions. For instance, a steady state is hardly observed in high-intensity exercise and mostly lasts for a shorter duration, causing data weight differences and prediction errors.

When using PPG signals to obtain heart rate data, the measurement primarily reflects pulse rate rather than heart rate [50]. While both the PPG pulse wave and the ECG R-wave capture the cyclical activity of the heart and are often considered equivalent, this equivalence does not universally apply across all measurement contexts [51]. For example, contemporary studies examining the validity of pulse rate variability (PRV) as a proxy for heart rate variability (HRV) frequently report conflicting results [52]. These inconsistencies arise from differences in the delay and frequency characteristics of the two signals, which can be influenced by factors such as neural activity, respiration, blood pressure, and other physiological variables. These differences also explain the divergence observed between HR data derived from PPG signals and ECG signals during high-intensity exercise in this study. Consequently, in specific scenarios, the pulse rate may not accurately reflect true heart rate variations, potentially compromising the precision of $\dot{V}O_{2}$ predictions. This issue is particularly pertinent when pulse rate, rather than heart rate, is used as an input parameter. Therefore, in the actual prediction and application processes, it is essential to consider these potential variabilities and adjust predictive algorithms according to different exercise intensities to ensure accuracy.

It is important to acknowledge the limitations of this study. Firstly, the participants included were only healthy adults with exercise experiences, so the conclusions cannot be extrapolated to the other populations with diseases or older age, including those who were inactive. Further studies can incorporate a wider range of subjects to determine whether the results would be different and may improve the application of the algorithm. Otherwise, because the type of exercise we chose was cycling, no results can be shifted to other physical activities with a greater range of arm and wrist motion, and the error that occurred in other exercises might lead to diametrically opposite results.

## 5. Conclusions

Overall, our findings indicate that the accuracy of HR monitors varies from light to severe exercise tests, depending on the sensor wearing positions. For $\dot{V}O_{2}$ estimation, the BPNN can reduce the error differences between HR monitors by incorporating information on RESP and $\dot{V}E$. Currently, ECG is the primary source of signals for developing algorithms that include HR as an input, whereas HR detection in wearable devices is based on PPG technology. Therefore, when developing algorithms that include HR, it is essential to consider and validate PPG technology to adjust the algorithm to real-world use and enhance measurement accuracy. This approach will provide valuable insights for future health monitoring, facilitating more straightforward and precise $\dot{V}O_{2}$ prediction and energy expenditure assessment.

## Figures and Tables

**Table 1:** Characteristics of participants.

Measurement	Male (n = 16)	Female (n = 12)
Age (y)	19 ± 0	19 ± 1
Body mass (kg)	70.7 ± 5.7	55.6 ± 3.9
Height (cm)	180.6 ± 5.1	166.3 ± 4.1
BMI (kg/m^2^)	21.7 ± 1.8	20.2 ± 1.5
Body fat percentage (%)	10.1 ± 2.7	18.8 ± 3.7
$\dot{V}O_{2}max$ (mL/(kg·min))	47.3 ± 4.7	39.5 ± 4.1

**Table 2:** HR accuracy data for the different exercise intensities.

Exercises and Devices	Mean Difference ± SD	Mean Relative Error ± SD	Mean Absolute Error ± SD	Concordance Correlation Coefficient (95% CI)	Pearson Correlation
45% $\dot{V}O_{2}max$					
Vantage	0.16 ± 3.79	0.06 ± 3.54	2.59 ± 2.40	0.96 (0.96–0.96)	0.96
Verity Sense	0.06 ± 2.99	0.00 ± 3.66	1.95 ± 2.00	0.98 (0.98–0.98)	0.98
60% $\dot{V}O_{2}max$					
Vantage	0.15 ± 3.55	0.09 ± 3.08	2.19 ± 2.18	0.95 (0.98–0.98)	0.98
Verity Sense	0.28 ± 4.36	0.18 ± 3.30	1.83 ± 2.75	0.97 (0.97–0.97)	0.97
75% $\dot{V}O_{2}max$					
Vantage	−0.04 ± 3.42	−0.08 ± 2.84	1.92 ± 2.09	0.99 (0.99–0.99)	0.99
Verity Sense	0.04 ± 2.90	0.01 ± 2.40	1.52 ± 1.85	0.99 (0.99–0.99)	0.99
95% $\dot{V}O_{2}max$					
Vantage	2.88 ± 13.29	1.64 ± 7.95	3.62 ± 7.26	0.85 (0.85–0.85)	0.86
Verity Sense	1.94 ± 10.42	1.17 ± 6.55	2.83 ± 6.06	0.91 (0.91–0.91)	0.91
110% $\dot{V}O_{2}max$					
Vantage	2.33 ± 11.62	1.35 ± 7.3	3.49 ± 6.56	0.81 (0.81–0.82)	0.82
Verity Sense	5.29 ± 17.56	3.18 ± 10.78	4.79 ± 10.17	0.54 (0.53–0.54)	0.57

**Table 3:** Comparison of accuracy between models in different exercises.

	Model	Devices	R^2^	MAE (mL/min)	MAPE (%)
45% $\dot{V}O_{2}max$	$\dot{V}O_{2HR}$	H10	0.27	204.85	21.83%
		Vantage	0.27	205.20	21.82%
		Verity Sense	0.26	204.91	21.81%
	$\dot{V}O_{2HR+RESP}$	H10	0.27	205.50	21.71%
		Vantage	0.26	206.35	21.90%
		Verity Sense	0.26	205.96	21.88%
	$\dot{V}O_{2HR+\dot{V}E}$	H10	0.85	88.98	9.29%
		Vantage	0.86	89.14	9.31%
		Verity Sense	0.86	89.01	9.30%
	$\dot{V}O_{2HR+RESP+\dot{V}E}$	H10	0.91	68.89	7.12%
		Vantage	0.91	68.50	7.00%
		Verity Sense	0.91	68.46	7.00%
60% $\dot{V}O_{2}max$	$\dot{V}O_{2HR}$	H10	0.52	244.11	22.39%
		Vantage	0.51	247.76	22.68%
		Verity Sense	0.50	251.31	22.96%
	$\dot{V}O_{2HR+RESP}$	H10	0.55	231.71	21.39%
		Vantage	0.54	235.89	21.72%
		Verity Sense	0.53	238.77	21.92%
	$\dot{V}O_{2HR+\dot{V}E}$	H10	0.89	120.45	11.10%
		Vantage	0.88	120.65	11.12%
		Verity Sense	0.88	120.75	11.12%
	$\dot{V}O_{2HR+RESP+\dot{V}E}$	H10	0.92	97.16	8.98%
		Vantage	0.92	100.54	9.22%
		Verity Sense	0.92	100.84	9.22%
75% $\dot{V}O_{2}max$	$\dot{V}O_{2HR}$	H10	0.74	242.43	19.29%
		Vantage	0.72	254.52	20.17%
		Verity Sense	0.73	249.75	19.81%
	$\dot{V}O_{2HR+RESP}$	H10	0.75	238.43	18.86%
		Vantage	0.72	250.88	19.78%
		Verity Sense	0.73	246.33	19.45%
	$\dot{V}O_{2HR+\dot{V}E}$	H10	0.92	139.40	11.14%
		Vantage	0.92	140.92	11.29%
		Verity Sense	0.92	140.55	11.25%
	$\dot{V}O_{2HR+RESP+\dot{V}E}$	H10	0.96	102.87	8.30%
		Vantage	0.95	105.48	8.53%
		Verity Sense	0.95	104.91	8.47%
95% $\dot{V}O_{2}max$	$\dot{V}O_{2HR}$	H10	0.73	317.35	23.48%
		Vantage	0.59	367.81	25.75%
		Verity Sense	0.62	355.60	25.38%
	$\dot{V}O_{2HR+RESP}$	H10	0.74	289.51	19.87%
		Vantage	0.58	345.66	22.30%
		Verity Sense	0.63	329.40	21.74%
	$\dot{V}O_{2HR+\dot{V}E}$	H10	0.88	215.10	15.37%
		Vantage	0.87	227.47	16.00%
		Verity Sense	0.87	223.89	15.87%
	$\dot{V}O_{2HR+RESP+\dot{V}E}$	H10	0.92	165.27	12.57%
		Vantage	0.88	200.27	14.31%
		Verity Sense	0.90	187.88	13.75%
110% $\dot{V}O_{2}max$	$\dot{V}O_{2HR}$	H10	0.67	216.25	18.44%
		Vantage	0.35	267.62	20.71%
		Verity Sense	0.06	315.36	22.62%
	$\dot{V}O_{2HR+RESP}$	H10	0.68	221.55	19.39%
		Vantage	0.37	276.45	22.01%
		Verity Sense	0.06	325.27	23.71%
	$\dot{V}O_{2HR+\dot{V}E}$	H10	0.82	162.12	13.63%
		Vantage	0.80	171.47	14.25%
		Verity Sense	0.78	176.67	14.43%
	$\dot{V}O_{2HR+RESP+\dot{V}E}$	H10	0.83	154.89	12.87%
		Vantage	0.79	174.08	14.02%
		Verity Sense	0.75	182.67	14.25%

**Table 4:** Comparison of accuracy between models in all exercises.

	Devices	R^2^	MAE (mL/min)	MAPE (%)	%-Increase in MAE
$\dot{V}O_{2HR}$	H10	0.65	278.66	28.78%	---
	Vantage	0.56	300.85	30.01%	7.96%
	Verity Sense	0.54	302.57	30.01%	8.58%
$\dot{V}O_{2HR+RESP}$	H10	0.66	276.90	29.29%	---
	Vantage	0.57	298.33	30.49%	7.74%
	Verity Sense	0.55	300.29	30.51%	8.45%
$\dot{V}O_{2HR+\dot{V}E}$	H10	0.84	188.76	17.98%	---
	Vantage	0.83	194.13	18.26%	2.85%
	Verity Sense	0.82	195.68	18.27%	3.67%
$\dot{V}O_{2HR+RESP+\dot{V}E}$	**H10**	**0.87**	**165.28**	**15.91%**	**---**
	Vantage	0.86	175.22	16.44%	6.02%
	Verity Sense	0.85	175.01	16.37%	5.89%

## Data Availability

The original contributions presented in this study are included in the article; further inquiries can be directed to the corresponding author.
